# Correction: Enantioselective C(sp^3^)–H bond functionalization enabled by Cp^*x*^M(iii) catalysis (M = Co, Rh, Ir)

**DOI:** 10.1039/d6sc90044e

**Published:** 2026-02-24

**Authors:** Shu-Bin Mou, Mu-Peng Luo, Feifei Fang, Shi Cao, Dong Wu, Shou-Guo Wang

**Affiliations:** a Computer Aided Drug Discovery Center, Zhuhai Institute of Advanced Technology, Chinese Academy of Sciences Zhuhai 519003 P. R. China wudong@ziat.ac.cn; b College of Chemistry and Environmental Engineering, Shenzhen University Shenzhen 518060 P. R. China shicaorganic@szu.edu.cn shouguo.wang@szu.edu.cn

## Abstract

Correction for ‘Enantioselective C(sp^3^)–H bond functionalization enabled by Cp^*x*^M(iii) catalysis (M = Co, Rh, Ir)’ by Shu-Bin Mou *et al.*, *Chem. Sci.*, 2026, **17**, 2990–3004, https://doi.org/10.1039/d5sc08394j.

The authors regret that three of the schemes in the published manuscript (Schemes 16, 20 and 21) had errors present in them. The corrected schemes are given below.

**Scheme 1:**
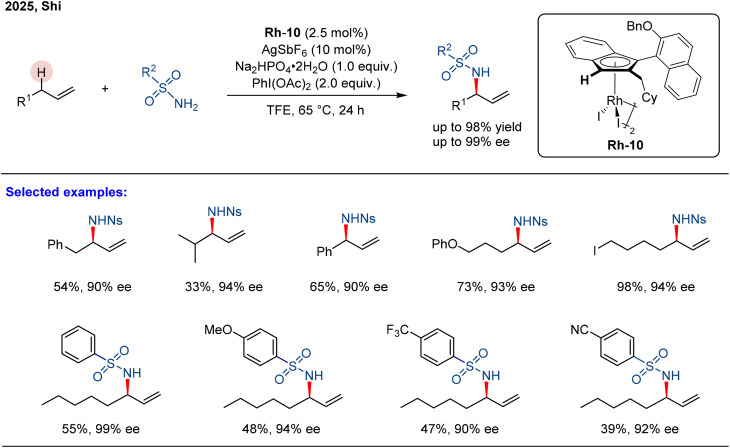
Rh(iii)-catalyzed asymmetric intermolecular allylic C–H amination of unactivated alkenes.

**Scheme 2:**
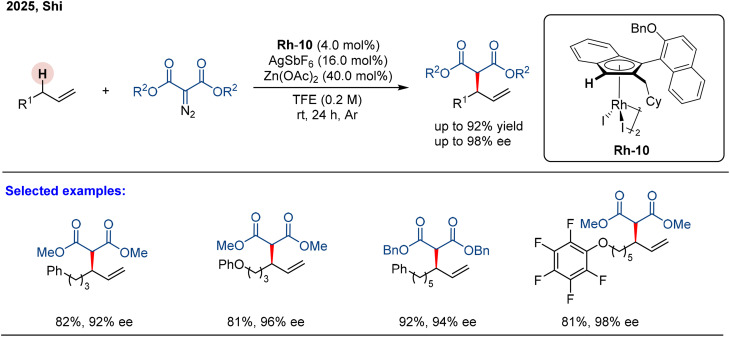
Rh(iii)-catalyzed asymmetric allylic C–H alkylation of α-olefins.

**Scheme 3:**
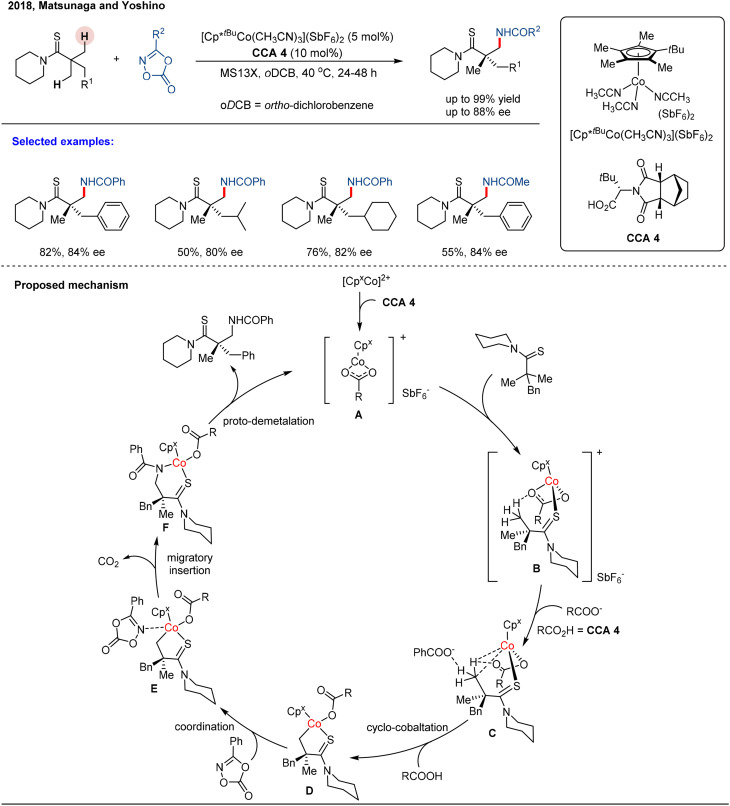
Co(iii)/**CCA**-catalyzed enantioselective C(sp^3^)–H amidation of thioamides.

The Royal Society of Chemistry apologises for these errors and any consequent inconvenience to authors and readers.

